# Chronic mesh infection complicated by an enterocutaneous fistula successfully treated by infected mesh removal and negative pressure wound therapy: A case report

**DOI:** 10.1097/MD.0000000000018192

**Published:** 2019-12-10

**Authors:** Hongquan Liu, Xiaochun Liu, Guofu Zheng, Bo Ye, Weiqing Chen, Hailiang Xie, Yunqiang Liu, Yi Guo

**Affiliations:** The Department of General Surgery, Ganzhou People's Hospital (The Affiliated Ganzhou Hospital of Nanchang University), Ganzhou, China.

**Keywords:** chronic mesh infection, enterocutaneous fistula, negative pressure wound therapy

## Abstract

**Rationale:**

Tension-free repair of inguinal hernia with prosthetic materials in adults has become a routine surgical procedure. However, serious complications may arise such as mesh displacement, infection, and even enterocutaneous fistula (EF). The management of chronic mesh infection (CMI) complicated by an EF is very challenging. A simple treatment of infected mesh removal and negative pressure wound therapy (NPWT) may cure the patients with EF with CMI.

**Patient concerns:**

A 75-year-old male patient underwent tension-free treatment for a bilateral inguinal hernia at a county hospital 10 years ago. Three months before admission, the right groin gradually formed a skin sinus with outflow of fetid thin pus, and it could not heal.

**Diagnoses:**

The patient was diagnosed preoperatively with mesh plug adhesion to the intestine, which resulted in low-flow EF combined with CMI.

**Interventions:**

The patient received a simple treatment mode consisting of an incision made from the original incision, but the new incision did not penetrate the abdominal cavity; treatment included resection of the fistula, removal of the mesh, repair of the intestine and local tissue, and continuous irrigation of vacuum sealing drainage (VSD) devices for NPWT.

**Outcomes:**

The infected mesh was completely removed. Five VSD devices were utilized to treat the EF and wound. The time from intervention to wound healing was 35 days, and follow-up for 6 months revealed no infection and no hernia recurrence in the right groin.

**Lessons:**

The NPWT is effective in treating CMI concomitant with EF and does not increase the risk of hernia recurrence.

## 1 Introduction

Patients and doctors are frustrated by the occurrence of chronic mesh infection (CMI) after tension-free inguinal hernia repair, which often means that the mesh needs to be removed to cure the infection.^[1]^ However, removing the infected mesh is difficult.^[2]^ The management of the infected mesh caused by enterocutaneous fistula (EF) involves both removing the infected mesh and curing the EF,^[3,4]^ as well as preventing the recurrence of hernia,^[5]^ which is a principal challenge for hernia surgeons.

In this study, we present a unique treatment for infected mesh removal and negative pressure wound therapy (NPWT) to successfully cure a case of EF with CMI that the patient experienced for 3 months, 10 years after tension-free inguinal hernia repair. Informed written consent was obtained from the patient for publication of this case report and accompanying images. The Medical Ethics Committee of Ganzhou People's Hospital approved the collection of case data for this clinical retrospective study.

## 2 Case presentation

A 75-year-old male patient without a history of diabetes or hormonal medication use underwent tension-free repair of bilateral indirect inguinal hernia at a county hospital 10 years ago with a Mard Mesh & Perfix plug (BARD Company, NJ, USA), which is made of polypropylene material. His postoperative recovery was uneventful, with no significant pain in the bilateral inguinal area and lower abdomen for an extended time, and no symptoms of systemic fever. However, 3 months before admission to our hospital, he experienced redness, swelling, and pain in the right groin, with high local skin tension, accompanied by systemic fever. He continued to receive treatment at that hospital and was subjected to local puncture through which a yellow, thin pus was extracted; the pus had a foul odor and was considered to be indicative of abscess formation. One 1 cm incision was made, and approximately 50 mL of pus was released. Antibiotics were administered intravenously, and his body temperature returned to normal the next day. Since then, the dressing was changed every day, but the amount of pus drained daily did not decrease, and occasionally, it appeared that gas was emanating from the wound; gradually, a sinus tract was formed. Even after treatment for nearly 3 months, the sinus tract did not heal.

**Figure 1:**
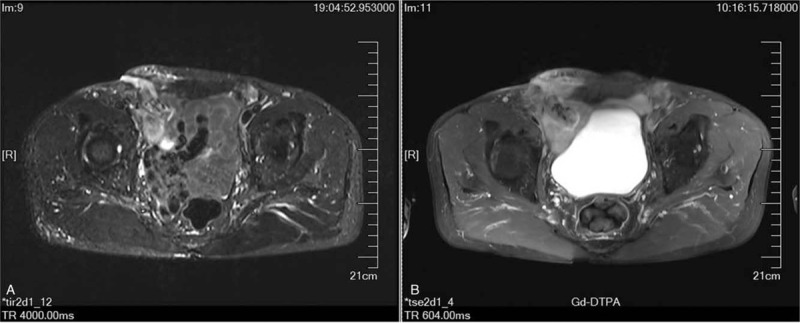
(A) Plain and (B) enhancement magnetic resonance imaging scan of the same plain (different time, different degree of bladder filling).

After admission, the patient showed good appetite, stool, urine, good nutritional status, and no fever. Surgical incision scars were observed on both sides of the groin and included redness and swelling on the right side, an ulcer of approximately 1 cm on the scar, and active purulent discharge. Forceps were used to probe the sinus tract, and the depth of the sinus tract was approximately 4 cm. The patient had no peritonitis symptoms except for tenderness in the right inguinal region. According to the medical records provided by the local hospital, the hernia sac was dissociated, which was followed by excision of the excess hernia sac, but the hernia sac was not closed. Instead, the mesh plug was inserted into the abdominal cavity through the hernia ring opening; then, the plug and peritoneum were stitched together with nonabsorbable suture to close the hernia sac. Routine blood examination on admission showed a normal white blood cell count and an abnormal increase in C-reactive protein (67.5 mg/L); moreover, *Streptococcus hemolyticus* was cultured from the wound secretion. Plain and enhanced magnetic resonance imaging scans of the lower abdomen suggested right inguinal mesh and soft tissue infection as well as sinus tract formation (Fig. 1). On imaging, adhesion was observed between the mesh plug and intestine, but no abnormal features of infection, such as an abscess in the abdominal cavity, were observed. After oral administration of methylene blue for 3 hours, the wound dressing pus was pale blue (unfortunately, no photos were obtained). Based on the comprehensive medical history, we diagnosed the patient with mesh plug adhesion to the small intestine resulting in low-flow EF combined with CMI.

The treatment was as simple as possible. Instead of entering the abdominal cavity, the infected mesh plug was removed through the original incision scar, and then, the isolated small intestine, surrounding scar tissue and aponeurosis of obliquus externus abdominis were repaired with absorbable suture, leaving a gap. The foam dressing (made of polyvinyl alcohol) of the vacuum sealing drainage (VSD; Wuhan VSD Medical Science & Technology Co, Ltd, Wuhan city, China) was inserted into the gap and covered the incision and the NPWT was performed with the pressure of 125 to 300 mm Hg in continuous mode.

**Figure 2:**
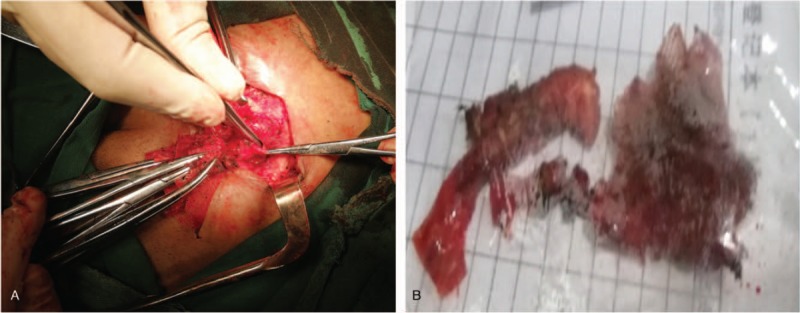
(A) The hole probed with forceps was perforated and thickened intestinal wall where adhesion to the mesh plug. (B) The removed integral infected mesh plug.

After 3 days of intestinal preparation, an additional 6 cm was cut from the original incision scar and the surrounding adhesion was dissociated. It was found that the plain mesh was closely attached to the transverse abdominal fascia and adhered to the mesh plug below. An abscess cavity had formed around the mesh plug from which thin pus was extracted. The area surrounding the mesh plug was relaxed, but the basilar part of the mesh plug was attached to the small intestine, where a small amount of digestive fluid was intermittently discharged. An EF was confirmed intraoperatively (Fig. 2A: The hole probed with forceps was perforated, and a thickened intestinal wall with adhesion to the mesh plug was observed). We continued to dissociate the base of the plug and removed the infected mesh integrally (Fig. 2B).

**Figure 3:**
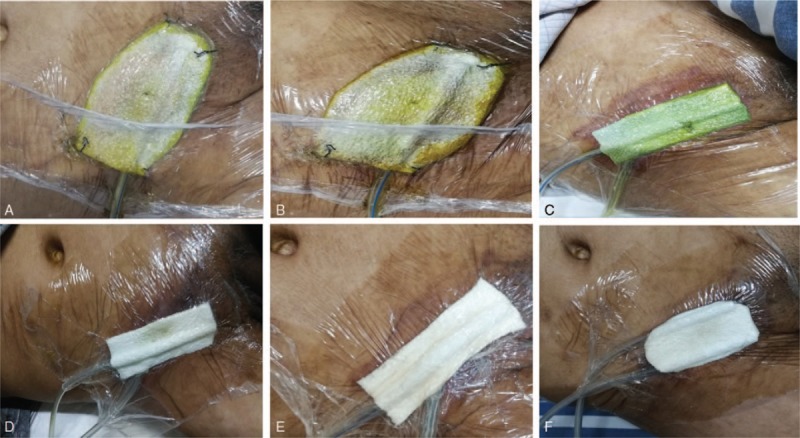
(A) The condition of the 1st VSD at 1st day postoperatively. (B–F) The respective conditions of 5 vacuum sealing drainage devices on the 3rd day after installation. The color of the foam dressing gradually changed from dark green to light till the green was completely faded.

**Figure 4:**
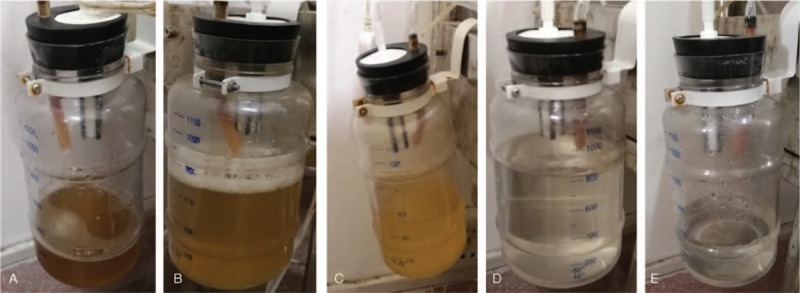
(A–E) The condition of drainage fluid on the 3rd day after each vacuum sealing drainage device replacement. The turbidous drainage fluid gradually became clear.

**Figure 5:**
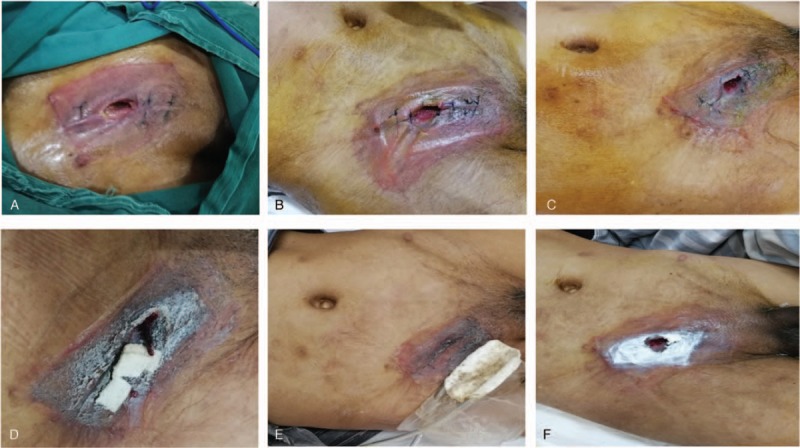
(A–E) The wound condition after each removal of vacuum sealing drainage (VSD). The fistula gradually shrinked and finally closed. (F) The wound condition protected by zinc oxide ointment before covering VSD.

Then, the local infected tissue was eliminated, the separated bowel was repaired with absorbable suture, and the wound was repeatedly washed with hydrogen peroxide and saline. The scar tissue was sutured, and absorbable suture was used on the aponeurosis of obliquus externus abdominis, leaving a space of approximately 2 cm for the insert of the VSD foam dressing. Most of the incisions were sutured, and the incision was then covered with foam dressing. The wound surface was continuously rinsed with saline for NPWT; the daily usage of saline was 500 to 1000 mL. On the first postoperative day, the VSD foam dressings appeared yellowish green (Fig. 3A). According to the instructions for use, each VSD device was used for 1 week. The color of the VSD dressing (Fig. 3B–F) and drainage fluid and the amount of drainage fluid (Fig. 4A–E) were observed on the third day. The wound conditions were observed after each VSD device change (Fig. 5A–E). When the VSD device was replaced, zinc ointment was applied to the wound for protection (Fig. 5F). Five VSD devices were used before wound healing (Fig. 5E), and the time from intervention to wound healing was 35 days.

Postoperatively, the sensitive antibiotic levofloxacin was used to treat the infection for 10 days. The patient had no symptoms of abdominal pain, peritonitis, or fever except for the pain and discomfort from the surgical incision. The white blood cell counts were normal in a repeat examination. The patient had flatus on the 1st postoperative day and defecation on the 2nd day. On the 2nd day, he received nutrition support with oral enteral nutritional suspension instead of intravenous nutrition support. The daily amount of fluid leakage in this patient was approximately 50 mL, which indicated a low-flow fistula, and no gastrointestinal digestive fluid inhibitors such as somatostatin were used. The support time of oral enteral nutritional suspension was 1 month.

**Figure 6:**
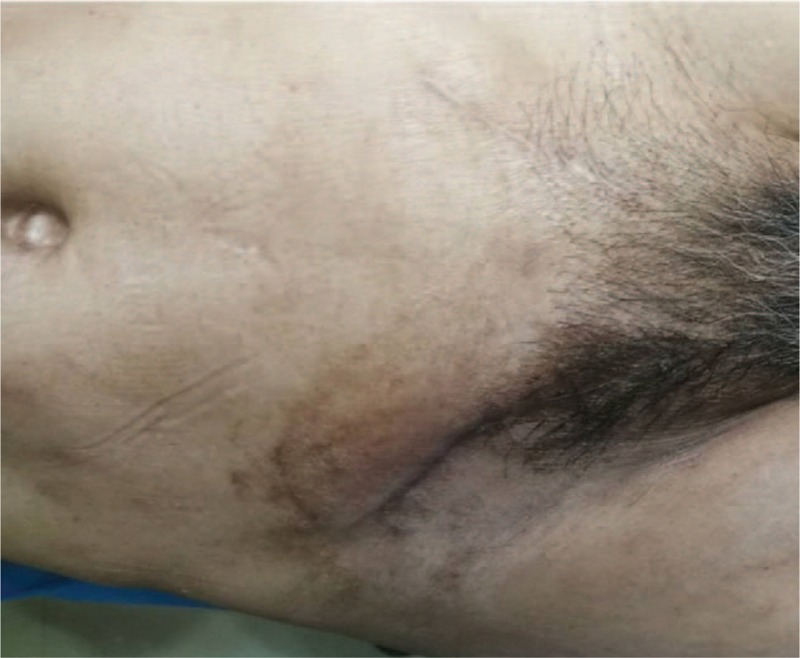
Six months of follow-up showed no bilateral inguinal infection and no recurrence of hernia.

Six months of follow-up (Fig. 6) revealed no bilateral inguinal infection and no recurrence of hernia.

## 3 Discussion

The merit of the treatment in this case is that a simple surgical approach was used to address the complex clinical issues.

Due to the increasing application of prosthetic materials, especially polypropylene mesh, in tension-free hernia repair, more and more cases of CMI have been reported.^[6–11]^ The clinical features of this case were that the mesh directly contacted the bowel after tension-free repair of bilateral inguinal hernia, and an EF occurred on the right side, which was accompanied by mesh infection and local abscess formation. Similar reports have been published,^[3]^ but we used unique interventions.

Mesh infection may be caused by many factors, such as nonstrict aseptic surgery,^[12]^ the suture fixation mode of the mesh,^[13]^ and the type of mesh.^[13–15]^ In addition, due to mesh migration or direct contact with the bowel, the mesh was infected by EF after mesh adhesion and corrosion of the bowel.^[3,4,16,17]^ Whether the contralateral side of this case has EF accompanied by mesh infection or not remains to be determined during long-term clinical follow-up.

The treatment of CMI is complex, which often means that the mesh needs to be removed completely, especially the initially used heavy weight and small pore polypropylene mesh. Once infected, the treatment method of retaining the mesh cannot cure the infection.^[1,8]^ Some researchers compared the effects of complete and partial removal of the infected mesh and found that maximal mesh removal is mandatory to cure the infection.^[18]^ Most people chose to remove the mesh through the original incision scar, through which the infected tissue, sinus tract, and fistula can be completely removed, and the eroded peripheral organs such as the intestines, bladder, and vagina, among others, could be explored and treated accordingly.^[3,4,19]^ There have also been reports of mesh removal through the preperitoneal space under endoscopy.^[20]^

The treatment of EFs also presents significant challenges, and there is currently a lack of high-quality evidence supporting any particular regimen of care; additionally, the surgeon is required to exercise skillful judgment in treating these individuals.^[21]^ Definitive surgery is not usually performed in the early stage of EF because surgery often does not cure EF, but instead, exacerbates intraabdominal infection.^[22]^ Since the emergence of NPWT,^[23,24]^ negative pressure drainage treatment for various infected wounds including abdominal infected wounds has been gradually applied in clinical practice and has achieved good results.^[25–28]^ Continuous NPWT can maintain the clean nature of the EF wound surface, promote formation of local granulation tissue,^[23]^ and even resolve EF.^[29,30]^ Negative pressure drainage does not increase the incidence of EF.^[31]^

One of the therapeutic objectives for this case was to cure EF and heal the infected wound. The treatment modes we chose were resection of the fistula, removal of the abscess, separation of the adhesion, removal of the infection mesh, repair of the intestinal adhesion leakage, suture and repair of local tissue, and finally, the continuous irrigation and NPWT of the VSD. The reasons are discussed below. First, the patient had local abdominal wall infection for more than 3 months, without local manifestations of peritonitis with abdominal cavity infection, and no systemic infection or fever symptoms. The magnetic resonance imaging results indicated abdominal wall mesh infection with sinus tract formation and no intra-abdominal infection-related lesions (Fig. 1). Second, the formation time of EF was more than 3 months, and the local lesions were basically stable. Third, the most important reason for not entering the abdominal cavity during the operation was so that the contamination of the abdominal cavity by infected materials at the abdominal wall could be avoided; this would prevent postoperative abdominal infection, abscess formation, intestinal adhesion, and other complications. Fourth, continuous washing and NPWT had good treatment effects on the EF^[30]^ and wounds caused by infected mesh.^[32,33]^ Finally, under the premise of full NPWT, patients could be given enteral nutrition without the use of somatostatin, which had almost no negative impact on patients’ overall nutritional status and could further promote wound healing.^[34]^

Preventing hernia recurrence was another important therapeutic objective for the patient. There were 2 different opinions about whether the removal of the infected mesh leads to the recurrence of hernia or not. One opinion was that hernia might be recurred after removal of the infected mesh.^[35]^ Therefore, some people thought that different methods could be used, such as after removing the infected mesh and at same time inserting another new mesh again^[35]^ or partial mesh removal for the treatment of CMI,^[18]^ some researchers suggested to repair the hernia using biosynthetic absorbable mesh after the infected mesh removal.^[36]^ Another opinion suggested that the removal of the infected mesh could not result the hernia recurring.^[8]^ The long-term presence of polypropylene meshes in the body always produces a foreign body reaction, attracts local inflammatory cells, and induces local fibrous connective tissue hyperplasia and collagen formation.^[37]^ These pathological reactions resulted in scarring in deep tissue of the abdominal wall. These scars were tough, as strong as the transverse fascia, and they were the patient's own tissue, which could be stitched together with absorbable suture to repair the defect by removing the infected mesh and without increasing the risk of infection. After the infectious material was removed, the proliferating tenacious tissue was sutured with absorbable sutures, and a small incision was reserved to insert foam dressing for continuous flushing and NPWT of the deep infected wound. NPWT is beneficial to the growth of granulation tissue. With the closure of the intestinal fistula and the growth of granulation tissue and early scar tissue, the wound defect was eventually naturally restored after the mesh was removed; recurrence of hernia was not observed.^[8]^

This treatment mode increased the length of stay of the patient but reduced the operative risk and the financial burden.

That no contrast radiography was performed on the fistula was a limitation in the preoperative examination of this case. Although clinical evidence supported the presence of a low-flow small bowel fistula, the lack of contrast radiography was still a major insufficiency.

Here, we provide a simple and unique method for the treatment of CMI with concomitant EF caused by mesh plug adhesion to the small intestine. This method can be applied in similar situations in the future.

## Author contributions

**Conceptualization:** Hongquan Liu, Xiaochun Liu.

**Data curation:** Guofu Zheng, Weiqing Chen, Hailiang Xie, Yunqiang Lin.

**Formal analysis:** Xiaochun Liu.

**Investigation:** Weiqing Chen.

**Supervision:** Hongquan Liu, Bo Ye.

**Visualization:** Hailiang Xie.

**Writing – original draft:** Xiaochun Liu, Yunqiang Lin.

**Writing – review & editing:** Xiaochun Liu.

Xiaochun Liu orcid: 0000-0002-6324-2328.
